# Production and Bioactivity-Guided Isolation of Antioxidants with α-Glucosidase Inhibitory and Anti-NO Properties from Marine Chitinous Materials

**DOI:** 10.3390/molecules23051124

**Published:** 2018-05-09

**Authors:** Van Bon Nguyen, Thi Hanh Nguyen, Chien Thang Doan, Thi Ngoc Tran, Anh Dzung Nguyen, Yao-Haur Kuo, San-Lang Wang

**Affiliations:** 1 Institute of Research and Development, Duy Tan University, Da Nang 550000, Vietnam; bondhtn@gmail.com; 2 Department of Science and Technology, Tay Nguyen University, Buon Ma Thuot City 630000, Vietnam; nguyenhanh2208.tn@gmail.com (T.H.N); doanthng@gmail.com (C.T.D); tranngoctnu@gmail.com (T.N.T); 3 Department of Chemistry, Tamkang University, New Taipei City 25137, Taiwan; 4 Institute of Biotechnology and Environment, Tay Nguyen University, Buon Ma Thuot City 630000, Vietnam; nadzungtaynguyenuni@yahoo.com.vn; 5 Division of Chinese Materia Medica Development, National Research Institute of Chinese Medicine, Taipei 11221, Taiwan; kuoyh@nricm.edu.tw; 6 Life Science Development Center, Tamkang University, New Taipei City 25137, Taiwan

**Keywords:** antioxidants, anti-inflammation, chitin, exopolysaccharides, homogentisic acid

## Abstract

Natural and bioactive products have been of great interest due to their benefit as health foods and drugs to prevent various diseases. The aim of this study is to efficiently reuse marine chitinous materials (CMs), abundant and low-cost fishery by-products, for the bio-synthesis, isolation, and identification of antioxidant compounds possessing some other beneficial bioactivities. *Paenibacillus* sp. was used to convert CMs to antioxidants. Among various tested CMs, squid pen powder (SPP) gave the best results when used as the sole carbon/nitrogen source. Fermented SPP (FSPP) had comparable antioxidant activity (IC_50_ = 124 µg/mL) to that of α-tocopherol (IC_50_ = 30 µg/mL). The antioxidant productivity increased 1.83-fold after culture optimization. The use of multiple techniques, including Diaion, silica, and preparative HPLC columns coupled with a bioassay resulted in the isolation of two major antioxidants characterized as exopolysaccharides and homogentisic acid. These isolated compounds showed great maximum activity and low IC_50_ values (96%, 30 µg/mL and 99%, 5.4 µg/mL, respectively) which were comparable to that of α-tocopherol (95%, 24 µg/mL). The crude sample, fractions, and isolated compounds also demonstrated α-glucosidase inhibition and anti–inflammatory activity. Notably, homogentisic acid was found as a non-sugar-based moiety α-glucosidase inhibitor which show much higher inhibition (IC_50_ = 215 µg/mL) than that of acarbose (IC_50_ = 1324 µg/mL) and also possessed acceptable anti–inflammatory activity (IC_50_ = 9.8 µg/mL). The results highlighted the value of using seafood processing by-products, like squid pens, for the production of several compounds possessing multi-benefit bioactivities and no cytotoxicity.

## 1. Introduction

Living organisms are negatively affected by free radicals [1]. Synthetic and natural antioxidants have been used to prevent damage, but synthetic antioxidants may result in liver damage and carcinogenesis [2]. Therefore, the discovery of new, abundant, low-cost, active and green production methods for natural antioxidant compounds is needed.

Antioxidants can be obtained from many sources, such as microbial conversion [3], higher plants [4,5,6], marine products [7] or synthesis [8]. Of these, microbial conversion has provided a very large number of novel active products and is of great interest due to the valuable characteristics of the microbes, such as fast growth and synthesis of various bioactive compounds under different conditions, as well as the advantages of its green production process, such as low-cost materials, mild fermentation, and no environmental pollution [9,10,11].

Antioxidant compounds, especially exopolysaccharides (EPSs), have been extensively produced from media rich in sugars, including sucrose, glucose, or galactose via conversion by fungi, such as *Fusarium solani* SD5 [1], *Aspergillus* sp. Y16 [12] *Pleurotus sajor-caju* [13], *Cordyceps militaris* SU5-08 [14], *Fomes fomentarius* [15], *Agrocybe cylindracea* [16], *Tremella fuciformis* [17], *Collybia maculate* [18], *Tremella mesenterica* [19], and *Cordyceps jiangxiensis* [20]. In contrast, there are few reports on EPS production with antioxidant properties from marine chitinous materials via bacterial conversion [3]. Similar to EPS antioxidants, there are few reports on homogentisic acid induced via bacterial conversion [11].

CMs have been widely obtained from fishery by-products as waste, including squid pens, crab shells, shrimp shells, and shrimp heads. Conventionally, these abundant and low-cost materials are used for chitin production by using strong acids and bases for demineralization and deproteinization, respectively. These chemical procedures cause several reported drawbacks involved in environmental pollution. In recent years, CMs have much interest for production of additional highly bioactive products, including antioxidants via microbial conversion [3,11].

In previous studies, *Paenibacillus* sp. TKU042, a bacterium isolated from Taiwanese soils, could produce active α-glucosidase inhibitors and antioxidants when cultivated in a commercial medium like nutrient broth [21]. Meanwhile, squid pen powder (SPP) was found to have potential as a material for antioxidant production by *Paenibacillus* sp. TKU036 [11]. Moreover, the genus of *Paenibacillus* rarely synthesizes products harmful to humans [1,7,8]. Therefore, the aims of this study are investigation of the efficient reuse of CMs like fishery processing by-products for antioxidant production via *Paenibacillus* fermentation, as well as the isolation, identification, and detection of other beneficial bioactivities of the isolated antioxidant compounds.

To achieve this, various chitinous materials and nutrient broth were used as the sole C/N sources for microbial conversion. A total of nine bacterial strains, including five strains of *Paenibacillus* and four strains of chitinolytic and/or proteolytic enzyme-producing bacteria, were used to ferment squid pens, the fishery processing by-product with the greatest potential. Optimization of cultivation conditions and partial purification of the fermented product were carried out in order to enhance antioxidant productivity and the isolation of the antioxidant compounds. The cytotoxicity, anti- inflammatory activity, and α-glucosidase inhibition of crude sample, fractions, and isolated compounds were also explored in this study. 

## 2. Results and Discussion

### 2.1. Screening Suitable C/N Source for Antioxidant Production by Paenibacillus sp. TKU042

**Figure 1:**
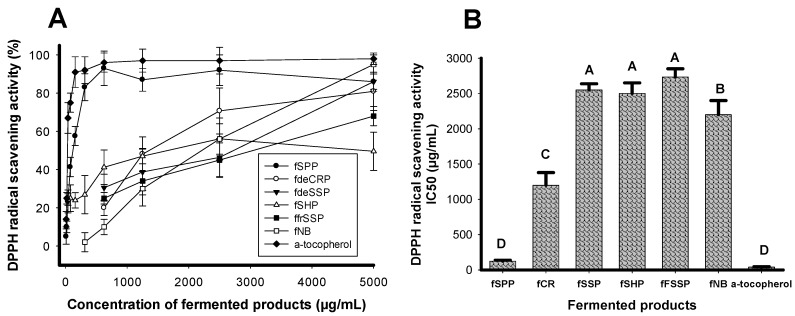
Antioxidant activity of CMs and nutrient broth fermented by *Paenibacillus* sp. TKU042. The culture supernatants were harvested after three days of fermentation. Tested antioxidant activity was then expressed as percentage (**A**) and IC_50_ (**B**) values; least significant difference (α = 0.1) = 300.77, coefficient of variation = 7.439375, triplicates for each experiment (n = 3), and means of IC_50_ values with the same letter are not significantly different in comparison based on t-test ranking.

Five kinds of chitinous materials, including SPP, demineralized crab shell powder (deCSP), shrimp head powder (SHP), demineralized shrimp shell powder (deSSP), and fresh shrimp shell powder (frSSP), were used to test antioxidant production via *Paenibacillus* sp. TKU042, a bacterium previously found to produce antioxidants when cultured in nutrient broth (NB) [21]. NB was also used in this experiment for comparison. As shown in Figure 1, SPP showed maximum activity and a low IC_50_ value at 94% and 142 µg/mL, respectively. Fermented SPP antioxidants had comparable activity to those of α-tocopherol with the same grade (D level) based on *t*-test ranking (Figure 1B). As a result, SPP was chosen for further investigation in subsequent experiments. 

Squid pens, an abundant and low-cost seafood processing by-product [22], have been used for the production of numerous bioactive materials by fermentation. SPP has been extensively studied for the production of chitin and chitosan [23], chitooligomers, and enzymes [23,24], exopolysaccharides [3,25,26], and biosorbents [27,28]. However, aside from the previous studies [3,11], few reports have focused on using SPP to produce antioxidant compounds. As such, it is of great economic value to investigate reusing this low-cost material for antioxidant production. 

### 2.2. Antioxidant Production by Paenibacillus and Chitinolytic and/or Proteolytic Enzyme-Producing Strains

To determine the best bacterial strain to induce active antioxidants with SPP, a total of nine bacterial strains were tested, including five strains of *Paenibacillus* and four stains of chitinolytic and/or proteolytic enzyme-producing bacteria. The results in Table 1 show that all tested strains of *Paenibacillus* had the same potency of antioxidant production. Fermented SPP showed maximum activity (83–94%) and IC_50_ values (124–169 µg/mL) that were comparable to α-tocopherol (95%, 30 µg/mL). Conversely, the four chitinolytic and/or proteolytic enzyme-producing bacterial strains demonstrated weak antioxidant production with low maximum activity (44–57%) and high IC_50_ values (2600–3500 µg/mL).

SPP, a fishery processing by-product, was reported to contain 40% chitin and 60% protein [23]. This material was used as the sole C/N source for fermentation in this experiment. Four chitinolytic and/or proteolytic enzyme-producing bacterial strains were used to ferment SPP. Unexpectedly, all tested strains showed weaker activity than *Paenibacillus*. Among the over 350 bacterial strains isolated from Taiwanese soil, *Paenibacillus* sp. TKU036 was reported to have the highest antioxidant activity at 85% [11]. However, *Paenibacillus* sp. TKU042 showed even higher activity (94%) than that of the TKU036 strain, and also showed the highest activity among the nine strains tested in this study. As such, this bacterium was chosen for further investigation.

### 2.3. Optimization of Cultivation Conditions for Enhancement of Antioxidant Production

#### 2.3.1. Optimization of Cultivation Time and Effect of Supplementary Air on Antioxidant Production

To determine optimal cultivation time, 100 mL of medium containing 1% SPP was fermented in a 250 mL Erlenmeyer flask for nine days. Cultivation was performed under two different sets of conditions: with supplementary air given once a day and with no supplementary air. Culture solutions were harvested daily and tested for antioxidant activity and bacterial growth.

**Figure 2:**
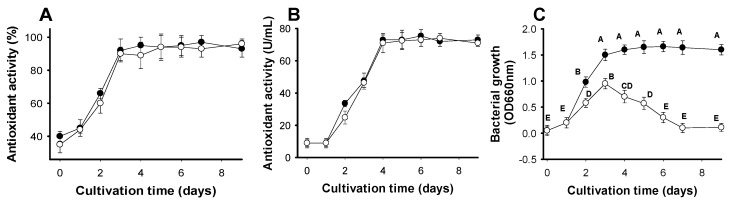
The effects of time and supplementary air on antioxidant activity (**A**), antioxidant productivity (**B**), and bacterial growth (**C**) via fermentation with *Paenibacillus* sp. TKU042, using SPP as the sole C/N source. (-●-): supplementary air once per day (-o-): no supplementary air; Least significant difference (α = 0.1) = 0.2658, coefficient of variation = 14.87304, triplicates for each experiment (n = 3), and means of IC_50_ values with the same letter are not significantly different in comparison based on *t*-test ranking.

Activity detected in the original culture supernatants (undiluted) are expressed as percent in Figure 2A. Under both sets of conditions, activity reached the maximum (approximately 94%) on day 3 with no increase thereafter. There was no significant difference in activity between the two cultures. To clarify the results, the original culture supernatants were diluted 25 times with methanol before activity was tested and expressed as U/mL (Figure 2B). Again, activity was equal under both sets of conditions. However, since the highest antioxidant production (≥75 U/mL) was achieved on day 4 of fermentation, four days was set as the optimal time for all subsequent experiments. 

Bacterial growth was also recorded daily (Figure 2C). The proportional correlation between antioxidant production and bacterial growth under both sets of conditions was only clearly observed from day 1 to day 3 of cultivation. After day 3, activity increased until day 4 and was stable thereafter. Conversely, bacterial growth did not increase after day 3 when supplementary air was given once a day and even dramatically decreased after day 3; the bacterial community died completely by day 7 of cultivation when no supplementary air was given. For convenience, fermentation without supplementary air was chosen for subsequent experiments. 

In the previous study [22], *Paenibacillus* sp. TKU042 also induced aGIs after four days of cultivation, but there were significant differences in aGI production depending on whether supplementary air was given. Fermentation with no supplementary air resulted in much greater activity (three-fold higher on day 4) than when supplementary air was provided. 

#### 2.3.2. Optimization of Parameters to Achieve Greater Antioxidant Productivity

**Figure 3:**
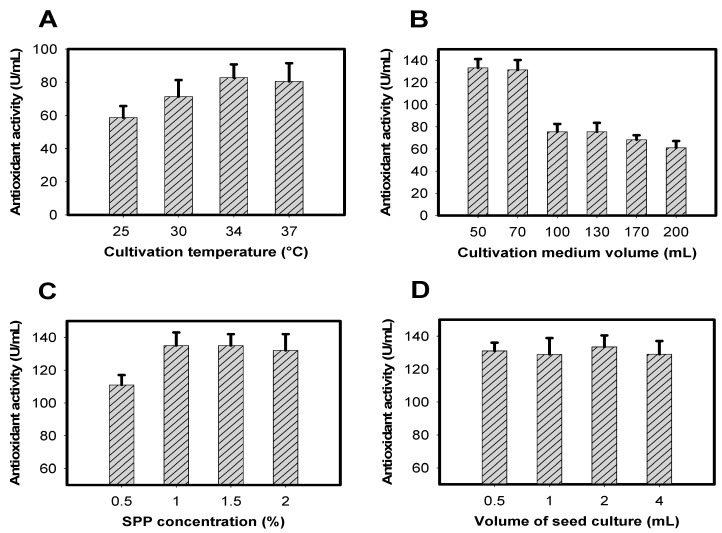
Effects of (**A**) culture temperature, (**B**) culture medium volume, (**C**) SPP concentration, and (**D**) bacterial seed volume on antioxidant production.

Other cultivation parameters, including temperature, medium volume, SPP concentration and volume of seed culture, were investigated to obtain greater antioxidant productivity. *Paenibacillus* sp. TKU042 induced highest antioxidant productivity (≥83 U/mL) at 34–37 °C (Figure 3A). In order to save energy, 34 °C was chosen while investigating the optimal cultivation medium volume. The results in Figure 3B show that cultivation of *Paenibacillus* sp. TKU042 in 50–70 mL of medium resulted in much higher antioxidant productivity (≥137 U/mL) than in 100–200 mL (≥60 U/mL). Taking the recovered culture supernatant volume into consideration, a medium volume of 70 mL was chosen for subsequent experiments (Figure 3C). Antioxidant production was highest with SPP concentrations in the range of 1–2%. SPP at 1% was suggested as the most suitable concentration for *Paenibacillus* sp. TKU042 to effectively induce antioxidants, and also saved on materials. Therefore, 1% SPP was chosen for subsequent experiments to investigate the effects of bacterial volume on antioxidant production. As shown in Figure 3D, this factor had no effect. No matter the amount of bacteria, antioxidant production remained the same (approximately 130 U/mL).

Overall, the optimal conditions for efficiently inducing antioxidants by *Paenibacillus* sp. TKU042 were 70 mL culture medium with an initial pH of 6.85, containing 1% SPP, 0.1% K_2_HPO_4_, and 0.05% MgSO_4_·7H_2_O in a 250 mL Erlenmeyer flask. Cultivation was performed at 34 °C and 150 rpm (shaking speed), with no supplementary air for four days. Activity increased 1.83-fold after optimization (75–137 U/mL).

In the previous study, *Paenibacillus* sp. TKU036 was also reported to produce antioxidants using the same C/N of SPP. However, SPP fermented by *Paenibacillus* sp. TKU036 gave the weaker activity (85%) than that of *Paenibacillus* sp. TKU042 (94%) after three days of fermentation. In addition, antioxidants produced from SPP via *Paenibacillus* sp. TKU042 could be obtained with greater volume scale (70 mL) in a lower cultivation temperature (34 °C) than those (50 mL, 37 °C) of *Paenibacillus* sp. TKU036 [11].

Antioxidants have been reported to be produced by some other bacterial strains, including *Monascus pilosus* fermenting potato dextrose broth [29], *Aspergillus usami* fermenting sesamin [30], *Bacillus subtilis* fermenting red bean [31], *Aspergillus awamori* and *Aspergillus oryzae* fermenting soybean [32], and numerous fungal strains fermenting medium rich in sugars, such as sucrose, glucose, or galactose [12,13,14,15,16,17,18,19,20]. Different to those of previous reports, *Paenibacillus* sp. TKU042 was used to ferment the cheaper and abundant N/C source like SPP. 

### 2.4. Isolation and Identification of Major Active Compounds from Fermented Product

Various techniques, including Diaion, silica opened columns, preparative HPLC column coupled with bioassay, and total sugars detection assays, were applied for partial purification and identification of the major active compounds from FSPP.

Several previous studies proved that *Paenibacillus* could induce EPSs [2,3,26,33,34,35]. Therefore, total sugar determination assay was also conducted to clarify whether the active components were EPS antioxidants or non-EPS antioxidants.

**Figure 4:**
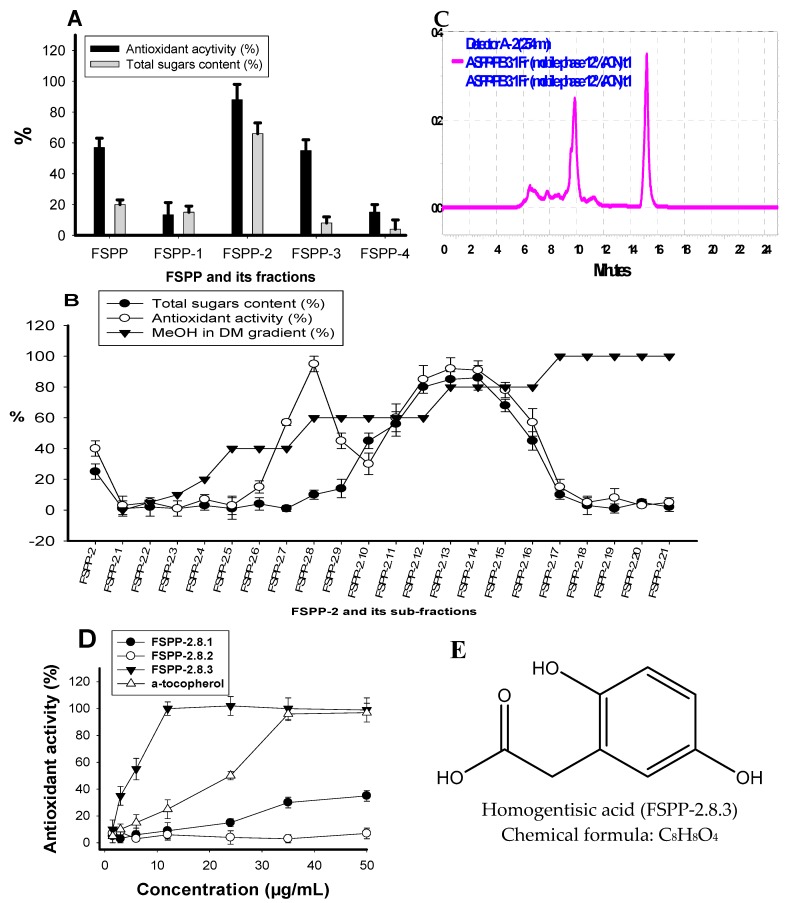
Antioxidant activity and total sugar content of fractions, sub-fractions and compounds after (**A**) Diaion opened column, (**B**) silica gel opened column, and (**D**) preparative HPLC column, respectively; (**C**) the HPLC finger prints of FSPP-2.8.1, FSPP-2.8.2, and FSPP-2.8.3 with the retention times at 6.5 min, 9.8 min, and 15.2 min, respectively; and (**E**) the chemical formula, structure of homogentisic acid.

Ten grams of FSPP was separated into five fractions: FSPP-1 (5 g), FSPP-2 (2.7 g), FSPP-3 (2 g), FSPP-4 (0.1 g), and FSPP-5 (0.05) were eluted with 1 L of distilled water, 30% EtOH, 70% EtOH, 95% EtOH, and 100% ethyl acetate, respectively. As shown in Figure 4A, FSPP-2 demonstrated the strongest activity, which was higher than that of the crude sample. Moreover, this fraction also showed the highest total sugar content (65%). Therefore, there is a high chance that the active compounds are EPS antioxidants. This potential fraction was further sub-fractionated by loading it onto a silica opened column and eluting continuously with methanol in dichloromethane (MeOH in DM) a gradient elution from 0/100–100/0. Twenty-one sub-fractions (FSPP-1 to FSPP-21) were collected and tested for antioxidant activity and total sugar content. The results illustrated in Figure 4B showed that two components (FSPP-2.8 and FSPP-2.13) possessed much stronger antioxidant activity than the FSPP-2 fraction, with values of 95%, 92%, and 25%, respectively. FSPP-2.8 contained very few sugars (total sugar content was ≤4%) while the total sugar content of FSPP-2.13 was 83%. Therefore, FSPP-2.13 and FSPP-2.8 were characterized as EPS and non-EPS antioxidants, respectively.

The non-EPS antioxidant (FSPP-2.8) was further purified by using preparative HPLC column. Three components of FSPP-2.8.1, FSPP-2.8.2, and FSPP-2.8.3 were obtained and tested for their activity. As presented in Figure 4D, FSPP-2.8.1 and FSPP-2.8.2 showed weak or no activity. In contrast, FSPP-2.8.3 demonstrated strong activity with maximum inhibition and IC_50_ values of 99%, and 8.1 µg/mL, respectively. To obtain greater purity of this potent component (FSPP-2.8.3), it was further separated by the same pre-HPLC column and recycled five times. Activity was enhanced 1.5-fold after this final purification step. The analysis of NMR data (1H NMR, 13C NMR) coupled with the comparison to that of the reported compound led to the identification of purified FSPP-2.8.3 as homogentisic acid (HGA) [11]. 

HGA (purified FSPP-2.8.3) was obtained as a white amorphous powder and its NMR chemical shifts were recorded as ^13^C-NMR data (100 MHz, MeOH-*d*_4_, *δ*_C_ ppm): 177.0, 151.6, 150.4, 124.3, 119.0, 117.4, 116.0, and 37.7; and ^1^H-NMR data (400 MHz, MeOH-*d*_4_, *δ*_H_ ppm): 6.62 (d, *J* = 8.4 Hz), 6.59 (d, *J* = 2.8 Hz), 6.53 (dd, *J* = 8.4, 2.8 Hz), and 3.50 (s, 2H). The chemical structure and formula of HGA are illustrated in Figure 4E. 

HGA is reported to possess valuable medicinal properties, such as antioxidant and anti-inflammatory activities [11], and plays a vital role in phenylalanine and tyrosine metabolism [36]. Recently, HGA was found to be an effective non-sugar-based α-glucosidase inhibitor [22]. Furthermore, this active compound was reported to be produced by only a few bacterial strains of *Aspergillus* [37], *Vibrio cholerae* [38], *Yarrowia lipolytica* [39], and *Paenibacillus* sp. TKU036 [11]. 

The EPS antioxidant FSPP-2.13 was further characterized their chemical bonds using enzymatic assays [3]. A total of 0.5 U/mL cellulase, pectinase, and α-amylase were used to hydrolyze FSPP-2.13. After 24 h of hydrolysis, the products (reducing sugars) were detected by measuring OD_540nm_ after mixing with dinitrosalicylic acid and heating at 100 °C for 10 min. Reducing sugars were only present in the FSPP-2.13 solution hydrolyzed by α-amylase. This result indicated that this EPS contains mostly α-1,4 glycosidic bonds rather than β-1,4 glycosidic bonds [3]. However, there was not enough proof to determine the sugar compositions and molecular weight of this EPS in this study. EPSs are also reported to possess other valuable bioactivities with promising applications in health food, pharmaceuticals, and other industries [25].

Antioxidant activity was dramatically increased by purification. After the final step of purification, activity increased 5.7-fold (FSPP-2.13) and 31.9-fold (purified FSPP-2.8.3) compared to the crude sample (FSPP). EPS antioxidants (FSPP-2.13) and HGA (purified FSPP-2.8.3) isolated from FSPP showed comparable or even much higher activity than that of α-tocopherol with IC_50_ values of 30, 5.4, and 24.1 µg/mL, respectively. The purification process and activity detection are summarized in Table 2.

### 2.5. Cytotoxicity, Anti-Imflammation and α-Glucosidase Inhibition of Crude Sample, Fraction, and Isolated Compounds

To confirm this fermented product and its isolated compounds as the good source of functional food with multi valuable and no toxicity, the cytotoxicity, α-glucosidase inhibition, and anti-inflammation were tested. As shown in Table 3, all the test samples, including crude sample, fraction, and isolated compounds, showed no cytotoxicity due to their great cell viability value of 91.42–103.05% at high concentration (80 µg/mL) of the tested samples.

α-Glucosidase inhibitory activity was commonly used for evaluation of in vitro anti-diabetic activity [21,22]. The samples were tested at various concentration then the activity was expresses as IC_50_ value. Acarbose, a commercial anti-diabetic drug was tested for the comparison. The isolated EPS show no activity (Table 3). However, the crude sample (FSPP), fraction (FSPP-2), and isolated compound (HGA) demonstrated stronger activity that that of positive control with the IC_50_ values of 275, 457, 215, and 1324 µg/mL, respectively. 

**Figure 5:**
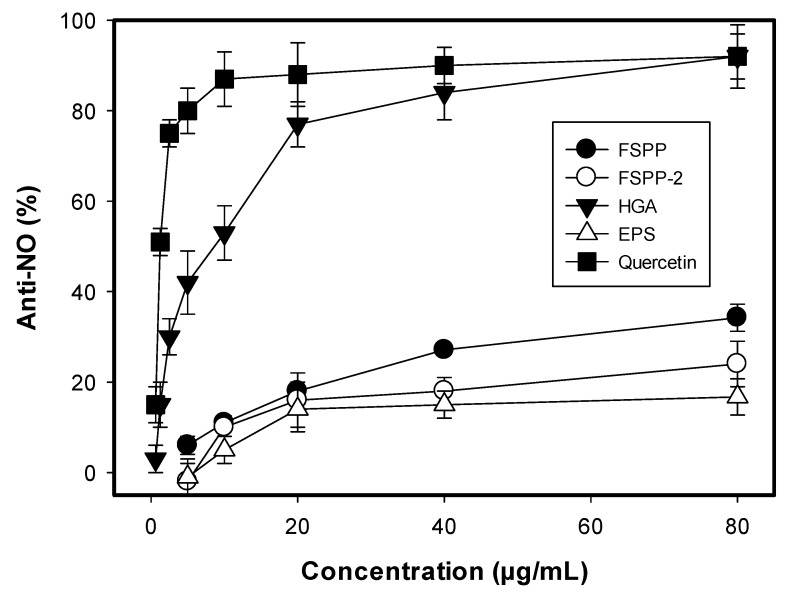
Anti-inflammation activity of FSPP, FSSP-2, and its isolated compounds. The cells of murine RAW 264.7 monocyte/macrophage were used as cell line model for testing anti-NO activity at the concentration range of 5–80 µg/mL of the samples and 0.625–80 µg/mL of positive control (quercetin).

It was reported that nitric oxide (NO) is considered as a mediator of pro-inflammatory activity involved in certain inflammatory disorders, such as pulmonary fibrosis, chronic hepatitis, and rheumatoid arthritis [40]. Anti-inflammation was determined by using LPS-stimulated RAW 264.7 cells as cell line models and presented in Figure 5. All tested samples showed activity with the value in the range of 16.7–92.1% at their concentration of 80 µg/mL. Of these, HGA demonstrated the strongest activity (92.1%). Quercetin, a positive anti-NO was tested for the comparison and showing higher activity (IC_50_ = 1.24 µg/mL) than HGA (IC_50_ = 9.8 µg/mL). These results suggest that FSPP could be developed as a potent functional food as it contains some compounds of HGA and EPSs possessing multi-benefit bioactivities and no toxicity.

In the previous study [24], chitooligomers with degrees of polymerization, varying from 3–9, possessing antioxidant and anti-inflammatory activities were reported, and production and isolation from chitosan hydrolyzed by a *Bacillus mycoides* chitosanase showed lower anti-inflammatory activity (with the IC_50_ values in the range of 76.27–82.65 µg/mL) than HGA isolated from FSPP in this study (IC_50_ = 9.8 µg/mL). HGA was also reported to be isolated and identified from SPP fermented by *Paenibacillus* sp. TKU036 in a recent report [11], showing the same anti-inflammatory activity (IC_50_ = 10.14 μg/mL) to that of isolated HGA in the current report. 

## 3. Materials and Methods

### 3.1. Materials

Squid pens, crab shells, and shrimp shells were acquired from Shin-Ma Frozen Food Co. (I-Lan, Taiwan). Shrimp head powder (SHP) was purchased from Fwu-Sow Industry (Taichun, Taiwan). Fresh shrimp shells were dried with lyophilization then ground to powder (FSSP). Demineralized crab shell powder (deCSP) and demineralized shrimp shell powder (deSSP) were prepared from crab shell and shrimp shell powders as per the methods described by Wang et al., 2006 [41]. Nutrient broth and 2, 2-diphenyl-1-picrylhydrazyl (DPPH) were purchased from Creative Life Science Co. (Taipei, Taiwan) and Sigma Chemical Co. (St. Louis City, MO, USA), respectively. The highest grade of solvents and common chemicals available were used. 

### 3.2. Bioactivity Assays

Antioxidant activity was determined using DPPH radical scavenging activity assay. One-hundred microliters (100 µL) of the samples (diluted 25 times with methanol) was mixed with 25 µL 0.75 mM DPPH in methanol in a 96-well plate. The optical density of the mixture solution was measured at 517 nm after being kept in the dark for 30 min [24]. Antioxidant activity was determined using the following formula:

(1) $$\mathrm{Antioxidant}\;\mathrm{activity}\;(\%)=\left(\frac{A-B}{A}\right)\times 100\%$$

where *A* stands for the optical density of the blank sample at initialization and *B* the tested sample solution at 30 min. The positive control was α-tocopherol dissolved in MeOH. IC_50_ value was defined as the concentration of an antioxidant that could reduce 50% of the DPPH purple color under assay conditions. The activity was also expressed as U/mL, where one U was defined as the volume of the sample required to inhibit 50% DPPH radicals [24].

Cytotoxicity and anti-inflammation assays were also carried out according to the methods described by Liang et al. (2016) [24] with modifications. The cytotoxicity of the samples was tested against murine RAW 264.7 monocyte/macrophage cell lines utilizing the MTT colorimetric protocol. Cells were cultivated in MEM medium. The samples with volume of 20 µL was placed in each well and incubated at 37 °C for 72 h after seeding cells in a 96-well microplate for 4 h, thereafter, 20 µL of MTT was added for 4 h. After the medium was washed off and DMSO (200 µL/well) was added to the microplate with mechanical shaking for 30 min. The formazan crystals were then re-dissolved and measured their absorbance at a wavelength of 550 nm. Quercetin was used as the positive control. α-Glucosidase inhibition was performed as the methods described by Nguyen et al., (2018) [9].

### 3.3. Optimization of Cultivation Conditions for Enhancement of Antioxidant Production 

#### 3.3.1. Optimization of Cultivation Time and Effect of Supplementary Air on Antioxidant Production

One gram of SPP was mixed with 100 mL of base mineral medium containing 0.1% K_2_HPO_4_ and 0.05% MgSO_4_·7H_2_O (initial pH 6.85) in a 250 mL Erlenmeyer flask. The prepared medium was fermented by *Paenibacillus* sp. TKU042. Fermentation was performed at 30 °C with a 150 rpm shaking speed for one to nine days. At the same time, cultivation was carried out under two sets of conditions: with supplementary air once per day or without any supplementary air. Air was supplemented by the methods described in detail by Nguyen et al., 2017 [22]. The culture supernatants were harvested daily and centrifuged at 4000 rpm for 20 min (used to detect activity) and at 500 rpm for 10 min (used to detect bacterial growth by measuring the optical density at 660 nm). 

#### 3.3.2. Optimization of Parameters to Achieve Greater Antioxidant Production

To obtain the highest antioxidant production, several parameters of fermentation were tested, including culture temperature (25 °C, 30 °C, 34°C, and 37 °C), culture volume (50 mL, 70 mL, 100 mL, 130 mL, 170 mL, and 200 mL), SPP concentration (0.5%, 1%, 1.5%, and 2%), and bacterial seed volume (0.5 mL, 1 mL, 2 mL, and 4 mL) (OD_660nm_ = 0.35). The antioxidant content of supernatants was tested on day 4, after centrifugation at 4000 rpm for 20 min.

### 3.4. Enzymatic Assays, Total Sugars, and Reducing Sugars Detection

Cellulase, pectinase and α-amylase assays and total sugars detection were performed, as described in the previous study [3]. Reducing sugar was determined following the methods described in the previous report [42].

### 3.5. Experimental Procedures for Partial Purification and Identification of Major Active Components from Fermented Product

Fermented SPP (FSPP, 10 g) was separated by a Diaion opened column (4 cm × 30 cm) into five fractions: FSPP-1 (5 g), FSPP-2 (2.7 g), FSPP-3 (2 g), FSPP-4 (0.1 g), and FSPP-5 (0.05) via elution with 1 L of distilled water, 30% EtOH, 70% EtOH, 95% EtOH, and 100% ethyl acetate, respectively. FSPP-2 (2.5 g) showed the highest activity and was further separated by loading onto a silica gel opened column (4 cm × 35 cm) then successively eluted by mobile phase with a gradient 0–100% (MeOH in DM, *v*/*v*) to give 21 sub-fractions. FSPP-2.8 (31 mg, eluted with 40% MeOH) and FSPP-2.13 (100 mg, eluted with 80% MeOH) showed potential activity. A pre-HPLC column [Cosmosil 5C18-AR-II column; 250 × 20 mm i.d. (Nacalai Tesque, Inc., Kyoto, Japan)] with a UV detection at 254 nm, a mobile phase of 12% acetonitrile, and a flow rate at 10 mL/min was used for further purification of FSPP-2.8. The active component (FSPP-2.8.3) obtained was cycled five times via the same column for greater purity, then underwent NMR analysis with 1H NMR and 13C NMR chemical shifts, using the previously-reported compounds for comparison. FSPP-2.13 was further confirmed as an exopolysaccharide by enzymatic assay, total sugar content, and reducing sugar detection.

### 3.6. Statistical Analysis

The differences between the mean values of antioxidant activity, enzyme inhibition, cell viability, and anti-NO (*p* < 0.01) were analyzed by using Statistical Analysis Software (SAS-9.4, provided by SAS Institute Taiwan Ltd., Minsheng East Road, Section 2, Taipei, Taiwan 149-8). All the tests were repeated in triplicate.

## 4. Conclusions

SPP was investigated as the best potential C/N source for antioxidant production by *Paenibacillus* sp. TKU042 among various chitinous materials. All tested *Paenibacillus* species showed the same manner of efficient conversion of SPP to antioxidants. Antioxidant production increased 1.83-fold after optimization of cultivation conditions. Antioxidant activity was also dramatically increased by purification. HGA (purified FSPP-2.8.3) and EPS antioxidants (FSPP-2.13) isolated from FSPP showed comparable, or even much higher, activity than that of α-tocopherol with IC_50_ values of 5.4, 30, and 24.1 µg/mL, respectively. The crude sample, fraction, and isolated compounds showed stronger α-glucosidase inhibition than that of acarbose. Anti–inflammatory activity was also detected. Of the tested samples, purified HGA demonstrated the most potent activity (92.1%, IC_50_ = 9.8 µg/mL). These results suggest that squid pens are valuable for bioactive materials production via bacterial conversion and this fermented product (FSPP) could be developed as healthy foods as it contains major compounds (HGA and EPSs) possessing multi-benefit bioactivities and no toxicity in in vitro testing.

## Figures and Tables

**Table 1:** Comparison of DPPH radical scavenging activity induced by *Paenibacillus* species and other chitinolytic and/or proteolytic enzyme–producing bacterial strains.

No.	Bacterial Strain	DPPH Radical Scavenging Activity	
		(%)	IC_50_ (µg/mL)
1	*Paenibacillus* sp. TKU042	94	124 ± 13.9 ^C^
2	*Paenibacillus* sp. TKU037	93	138 ± 16.2 ^C^
3	*Paenibacillus* sp. TKU036 (positive strain)	87	143 ± 10.3 ^C^
4	*Paenibacillus mucilaginosus* TKU032	83	169 ± 14.2 ^C^
5	Paenibacillus macerans TKU029	88	170 ± 20.3 ^C^
6	Bacillus sp. TKU004	56	2600 ± 346 ^B^
7	*Bacillus* cereus TKU028	45	3000 ± 288 ^AB^
8	*Bacillus mycoides* TKU038	44	3000 ± 173 ^AB^
9	*Lactobacillus* paracasei subsp *paracasei* TKU010	57	2700 ± 115 ^B^
Control (medium only)		40	3500 ± 289 ^A^
α-Tocopherol (commercial antioxidant)		95	30 ± 5.8 ^C^

The tested bacteria were cultivated in 100 mL medium containing 1% SPP at 30 °C, 150 rpm shaking speed for three days. Bacterial mass and medium residues were removed from culture medium solution by centrifugation at 4000 rpm. The supernatants were dried to powders then used for activity detection. Least significant difference (α = 0.1) = 538.97, coefficient of variation = 16.38701, triplicates for each experiment (n = 3), and means of IC_50_ values with the same letter are not significantly different in comparison based on *t*-test ranking.

**Table 2:** Enhancement of activity after partial purification of FSPP.

Purification Steps	Components	DPPH Radical Scavenging Activity		
		IC_50_ (µg/mL)	Specific Activity (U/mg)	Activity Folds Enhancement
Crude sample	FSPP	172 ± 15.2	5.8	1
Diaion opened column	FSPP-2	80 ± 7.9	12.5	2.2
Silica opened column	FSPP-2.8	19 ± 2.1	52.6	9.1
	FSPP-2.13	30 ± 1.9	33.3	5.7
preparative HPLC	FSPP-2.8.3	8.1 ± 0.71	123.5	21.3
Recycling	FSPP-2.8.3 (HGA)	5.4 ± 0.62	185.2	31.9
Positive control	α-tocopherol	24 ± 1.12		

**Table 3:** Cytotoxicity and α-glucosidase inhibition of FSPP, FSSP-2, and its isolated compounds.

Components	Cell Viability (%)	α-Glucosidase Inhibition IC_50_ (µg/mL)
FSPP	91.42 ± 3.87 ^B^	275 ± 11.2 ^b^
FSPP-2	103.05 ± 1.97 ^A^	457 ± 27.3 ^b^
FSPP-2.8.3 (HGA)	91.07 ± 2.37 ^B^	215 ± 15.3 ^b^
FSPP-2.13 (EPS)	95.80 ± 2.61 ^B^	—
Acarbose (positive aGI)		1324 ± 96.3 ^a^
*Least Significant Difference (α = 0.1)*	5.5628	269.31
*Coefficient of variation*	1.927574	15.66989

The cells of murine RAW 264.7 monocyte/macrophage were used as cell line model for testing cytotoxicity at the concentration of 80 µg/mL of the samples; the inhibition was tested against α-glucosidase from yeast; (—): no activity, triplicates for each experiment (n = 3), and means of cell viability IC_50_ values with the same letter are not significantly different in comparison based on *t*-test ranking.
